# Two new species and two new records of *Artabotrys* (Annonaceae) from Thailand

**DOI:** 10.3897/phytokeys.95.23434

**Published:** 2018-02-07

**Authors:** Junhao Chen, Piya Chalermglin, Richard M.K. Saunders

**Affiliations:** 1 School of Biological Sciences, The University of Hong Kong, Pokfulam Road, Hong Kong, P. R. China; 2 Agricultural Technology Department, Thailand Institute of Scientific & Technological Research, 35 Technopolis, Liap Khlong Ha Road, Khlong Luang District, Pathum Thani Province 12120, Thailand

**Keywords:** Annonaceae, *Artabotrys*, new records, new species, Thailand

## Abstract

Two new species of *Artabotrys* are described from Thailand. *Artabotrys tanaosriensis* J.Chen, Chalermglin & R.M.K.Saunders, **sp. nov.**, is similar to *A. oblanceolatus* Craib but differs in its symmetrical, cuneate or decurrent leaf base, externally distinct outer petal blades and claws, deltoid and undulate outer petal blades, rhomboid and undulate inner petal blades and shorter, subsessile and slightly beaked monocarps. *Artabotrys spathulatus* J.Chen, Chalermglin & R.M.K.Saunders, **sp. nov.**, is most similar to *A. tanaosriensis* but differs in having flat outer petal blades, broadly rhomboid outer petal claws, broadly spathulate and strongly concave inner petal blades and strongly beaked monocarps. Two new records for the Flora of Thailand are furthermore reported here: *A. punctulatus* C.Y.Wu ex S.H.Yuan and *A. byrsophyllus* I.M.Turner & Utteridge, which were previously confused with *A. aeneus* Ast and *A. grandifolius* King, respectively. A key to *Artabotrys* species indigenous to Thailand is provided here.

## Introduction

*Artabotrys* R.Br. is one of the largest palaeotropical genera in the Annonaceae, with ca. 105 species (Guo et al. 2017). The majority of the species occur in Asia, with only ca. 30 species in Africa (Mabberley 2008). *Artabotrys* species are woody climbers that are unique among climbing members of the Annonaceae in possessing specialised and persistent inflorescence hooks (Fig. 2B, D) to assist climbing; these hooks are derived from peduncles and bear flowers and fruits (Posluszny and Fisher 2000). Although these inflorescence hooks allow easy recognition of the genus, identification at the species level is often challenging (Turner 2009; Turner and Utteridge 2015). The leaf lamina is often decurrent to the petiole and the midrib is often raised on the adaxial surface of leaves. The inflorescences often bear several to many flowers, but are single-flowered in some species.

**Figure 1.**
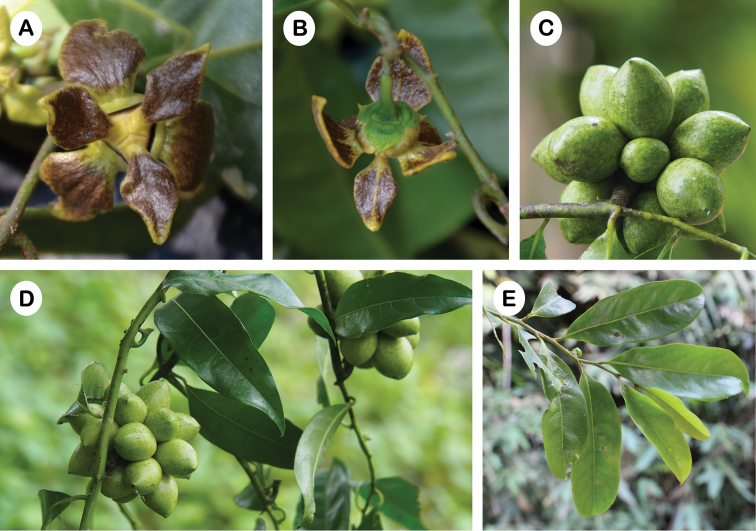
*Artabotrys tanaosriensis* **A** Flower with undulate petal blades and narrow constriction at the junction between petal blades and claws **B** Flower with rhomboid inner petal blades **C** Fruit with weakly beaked monocarps **D** Habit **E** Leaves (Photos: P. Chalermglin).

**Figure 2.**
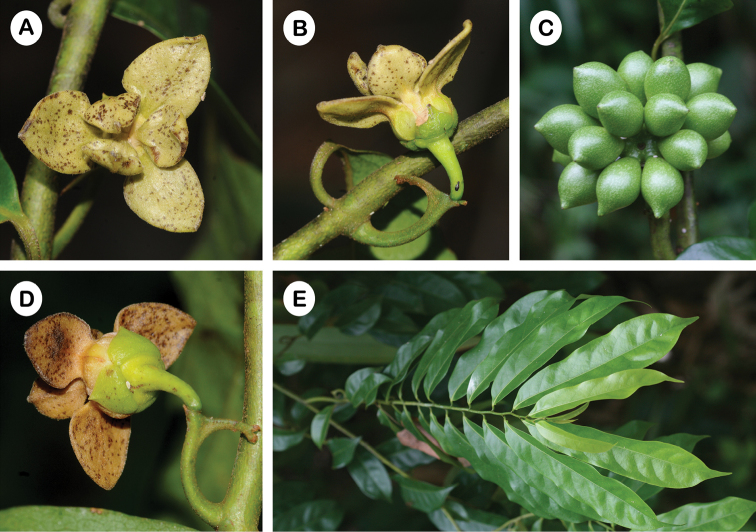
*Artabotrys spathulatus*. **A** Flower with flat outer petal blades and broadly spathulate and strongly concave inner petal blades **B** Inflorescence with hooked peduncle and flower with externally distinct inner petal claw and blade **C** Fruit with distinctly beaked monocarps **D** Flower with externally distinct outer petal blade and claw **E** Leaves and habit (Photos: P. Chalermglin).

The underlying floral Bauplan of *Artabotrys* species is rather uniform, with the exception of two African species, viz. *A. thomsonii* Oliv. (Le Thomas 1969) and *A. brachypetalus* Benth. (Robson 1960), which have been shown to be early divergent lineages in molecular phylogenetic reconstructions (Chen et al. unpubl.). Individual flowers possess one whorl of three sepals and two whorls of three petals each, with the sepals much smaller than the petals and with the two petal whorls subequal. Each petal has a distinct upper laminar blade and a basal concave claw, usually with a constriction at the junction between the two regions (Figs 1A, B, 2B, D). The inner petals are basally connivent, forming a dome that covers the reproductive organs, with three lateral apertures at the base of the dome, enabling pollinator access. The three inner petals abscise as a single unit after anthesis. The flowers are hermaphroditic, with numerous stamens and carpels. Each carpel contains two ovules with basal placentation. The fruits are apocarpous, with “monocarps” (derived from individual carpels after fertilisation) that are usually sessile or borne on very short stipes; the generic name is derived in part from the Greek word “*botrys*”, which alludes to its grape-like fruits.

Although *Artabotrys* is comparatively well-studied in Thailand, many names have been misapplied and several taxonomic and nomenclatural misunderstandings persist. Craib (1925) listed 11 species in his checklist of the Thai flora, viz. *A. brevipes* Craib, “*A. burmanicus* A.DC.” (a misapplied name, for which the name *A. siamensis* Miq. should be applied), *A. harmandii* Finet & Gagnep., *A. oblanceolatus* Craib, *A. hexapetalus* (L.f.) Bhandari (as “*A. odoratissimus* R.Br.”), *A. scortechinii* King, *A. siamensis*, *A. spinosus* Craib, *A. suaveolens* Blume (Blume), *A. vanprukii* Craib and *A. venustus* King. Chalermglin (2001, 2005) published a photographic guide to Thai Annonaceae that included 10 species, viz. “*A. burmanicus*” (a misapplied name), “*A. grandifolius*” (a misapplied name: see new records for Thailand below) *A. harmandii*, *A. hexapetalus*, “*A. oblanceolatus*” (a misapplied name), *A. siamensis*, *A. spinosus*, *A. suaveolens*, *A. vanprukii* and an unnamed species (which represents “true” *A. oblanceolatus*). More recently, Thongpairoj (2008) recognised 20 species in her unpublished PhD thesis, including four new records (*A. multiflorus* C.E.C.Fisch., *A. lowianus* Scort. ex King, *A. oxycarpus* King and *A. uniflorus* (Griff.) Craib) and six unnamed species. Insura (2009) subsequently recognised 15 species in his unpublished MSc thesis, of which four are new records, viz. “*A. aereus* Ast” (a misapplied name: see new records for Thailand below), “*A. blumei* Hook.f. & Thomson” (a misapplied name, for which the name *A. tipuliferus* I.M.Turner & Utteridge should be applied: Turner and Utteridge 2015), “*A. havilandii* Ridl.” (a misapplied name, for which the name *A. oxycarpus* should be applied: Thongpairoj 2008) and *A. sumatranus* Miq. Several species cited by these authors do not occur naturally in Thailand: *A. hexapetalus* is widely cultivated in Thailand but is likely to be of South Indian or Sri Lankan origin, for example; and Craib (1925) recorded *A. scortechinii* from Langkawi, an island that was previously under Siamese rule but now part of Peninsular Malaysia. No specimens of *A. scortechinii* from Thailand or Langkawi have been seen; *A. scortechinii* is likely to be endemic to Peninsular Malaysia and Singapore (Chen et al. in press). Additionally, three other species (*A. lowianus*, *A. sumatranus* and *A. vanprukii*) are poorly known owing to the absence of flowers and/or fruits in the type specimens, limited available material to assess variation and the problematic treatment of these taxa by previous authors. It is beyond the scope of this paper to clarify these taxonomic problems, however.

Examination of herbarium specimens and fieldwork in Thailand has revealed two new *Artabotrys* species, which are formally described here. In addition, two new records for Thailand are reported and past taxonomic errors rectified. A total of 15 species are recognised (excluding the aforementioned problematic taxa) and a key to the native species of *Artabotrys* in Thailand is provided.

## Materials and methods

The material studied comprises herbarium specimens of *Artabotrys* species from Thailand and neighbouring regions from the following herbaria: A, E, KUFF, KUN, L, NY, QBG, SING and US; high-resolution digital images of specimens (especially types) from JSTOR Global Plants (https://plants.jstor.org/) and other virtual herbarium websites; as well as fresh material collected during fieldwork in Thailand. Species delimitation was based on discontinuities (gaps) in morphological variation. The morphological gap is an indirect assessment of the underlying reproductive isolation because the lack of gene flow prevents two lineages from homogenising (Coyne and Orr 2004; Rieseberg et al. 2006). Morphological measurements were taken from dried herbarium specimens unless otherwise stated.

## Taxonomy

### Artabotrys tanaosriensis

Taxon classificationPlantaeMagnolialesAnnonaceae

J.Chen, Chalermglin & R.M.K.Saunders sp. nov.

urn:lsid:ipni.org:names:77175741-1

Fig. 1

#### Diagnosis.

Similar to *Artabotrys oblanceolatus* Craib except with cuneate or decurrent (vs. rounded) leaf base, symmetrical (vs. asymmetrical) leaf base, externally distinct outer petal blades and claws, deltoid and undulate (vs. ovate and flat) outer petal blades, rhomboid and undulate (vs. ovate and flat) inner petal blades, shorter monocarps (1.5–2 cm vs. 2–2.5 cm), slightly beaked (vs. sharply beaked) monocarps and monocarps with shorter stipes (ca. 2 mm vs. ca. 4 mm).

#### Type.

THAILAND: Phetchaburi Province, Kaeng Krachan National Park, Phanoen Thung Ranger Station, 1000 m elev., 25 Aug 2004, *I.C. Nielsen 1911* (holotype: L! [barcode L 3729228]; isotype: AAU, n.v.; SING! [barcode SING 0115634]).

#### Description.

Climbers, to 20 m in height. Twigs drying grey-brown, glabrous to sparsely hairy. Leaf laminas 9.5–15.5 cm long, 2.3–4.0 cm wide, oblong-elliptic, apex acuminate, base cuneate or decurrent, chartaceous, glabrous both ab- and adaxially; midrib glabrous to sparsely hairy abaxially, glabrous adaxially, prominent and raised on both surfaces; secondary veins 8–12 pairs per leaf, visible on both surfaces; tertiary venation reticulate, visible on both surfaces; petioles 2–5 mm long, ca. 1 mm in diameter, glabrous to sparsely hairy. Inflorescence hooks recurved, laterally compressed, with 1–3 flowers; flowering pedicels 7–9 mm long, ca. 1 mm in diameter, glabrous. Sepals ca. 4 mm long, 4–6 mm wide, triangular, subglabrous both ab- and adaxially, venation indistinct. Outer petals with externally distinct blades and claws; blades 10–12 mm long, 7–8 mm wide, deltoid, undulate, sparsely hairy both ab- and adaxially; claws ca. 4 mm long, 6–8 mm wide, broadly ovate, densely hairy abaxially, subglabrous adaxially. Inner petals with externally distinct blades and claws; blades 7–10 mm long, 4–5 mm wide, rhomboid, undulate, sparsely hairy both ab- and adaxially; claws ca. 6 mm long, ca. 4 mm wide, rhomboid, densely hairy abaxially, glabrous adaxially. Stamens ca. 90 per flower, ca. 1 mm long, ca. 1 mm wide; apex of connectives truncate. Carpels 24–30 per flower, ca. 1 mm long, ca. 1 mm wide; ovaries ovoid; stigmas ellipsoid, extending centrifugally. Fruiting pedicels 10–11 mm long, ca. 2 mm wide, glabrous. Monocarps 8–30 per fruit, 15–20 mm long, 9–13 mm wide, ellipsoid, glabrous, slightly beaked (beak less than 1 mm long), drying with longitudinal ridges, subsessile or with stipes up to ca. 2 mm long. Seeds 2 per monocarp, plano-convex, ca. 16 mm long, ca. 8 mm wide.

#### Phenology.

Flowering specimens collected in February and August; fruiting specimens collected in October.

#### Distribution and habitat.

So far only known from Thailand (Fig. 5), but possibly also occurring in the adjacent Tanintharyi National Park in Myanmar. Inhabits tropical rain forests on lateritic soil; 150–1000 m elev.

#### Etymology.

The specific epithet alludes to “Tanao Sri”, the Thai name of the Bilauktaung subrange of the Tenasserim Range where this species occurs.

#### Local name.

Karawek Tanao Sri.

#### Additional specimens examined (paratypes).

Thailand: Prachuap Khiri Khan Province, Hua Hin District, Huai Sat Yai, Pa La-U Village, 17 Oct 2016, *P. Chalermglin 591017* (BKF, HKU, L, QBG); Phetchaburi Province, Kaeng Krachan District, Huai Mae Phriang Village, 14 Feb 2015, *P. Chalermglin 580214* (BKF, HKU, L, QBG).

### Artabotrys spathulatus

Taxon classificationPlantaeMagnolialesAnnonaceae

J.Chen, Chalermglin & R.M.K.Saunders sp. nov.

urn:lsid:ipni.org:names:77175742-1

Fig. 2

#### Diagnosis.

Similar to *Artabotrys tanaosriensis* J.Chen, Chalermglin & R.M.K.Saunders except with flat (vs. undulate) outer petal blades, broadly rhomboid (vs. broadly ovate) outer petal claws, broadly spathulate and strongly concave (vs. rhomboid and undulate) inner petal blades and strongly beaked monocarps.

#### Type.

THAILAND: Krabi Province, Mueang Krabi District, Phruksa Sawan limestone hill, 100 m elev., 15 Mar 2015, *P. Chalermglin 580315* (holotype: BKF!; isotypes: L!, QBG!).

#### Description.

Climbers, to 20 m in height. Twigs drying brown, glabrous to sparsely hairy. Leaf laminas 9.6–18.1 cm long, 3–4.5 cm wide, oblong-elliptic, apex acuminate, base cuneate or decurrent, chartaceous, subglabrous both ab- and adaxially; midrib glabrous to sparsely hairy abaxially, glabrous adaxially, prominent and raised on both surfaces; secondary veins 6–14 pairs per leaf, visible on both surfaces; tertiary venation reticulate, visible on both surfaces; petioles 2–6 mm long, ca. 1 mm in diameter, glabrous to sparsely hairy. Inflorescence hooks recurved, laterally compressed, with 1 or 2 flowers; flowering pedicels ca. 10 mm long, ca. 1 mm in diameter, subglabrous. Sepals 4–5 mm long and wide, triangular, subglabrous both ab- and adaxially, venation indistinct. Outer petals with externally distinct blades and claws; blades 8–10 mm long, 7–8 mm wide, deltoid, flat, sparsely hairy both ab- and adaxially; claws ca. 5 mm long and wide, broadly rhomboid, densely hairy abaxially, glabrous adaxially. Inner petals with externally distinct blades and claws; blades 6–7 mm long, 4–5 mm wide, broadly spathulate, strongly concave, sparsely hairy both ab- and adaxially; claws ca. 5 mm long, ca. 3 mm wide, narrowly rhomboid, densely hairy abaxially, glabrous adaxially. Stamens numerous per flower, ca. 1 mm long, ca. 1 mm wide; apex of connectives truncate. Carpels ca. 30 per flower, ca. 1 mm long, ca. 1 mm wide; ovaries ovoid; stigmas ellipsoid, extending centrifugally. Fruiting pedicels unknown. Monocarps (in fresh material) up to 30 per fruit, 19–26 mm long, 14–18 mm wide, obovoid, strongly beaked (beak ca. 2 mm long), sessile. Seeds 2 per monocarp, plano-convex, 12–14 mm long, 9–11 mm wide.

#### Phenology.

Flowering and fruiting specimens collected in March.

#### Distribution and habitat.

So far only known from Thailand (Fig. 5). Inhabits tropical rain forests at the base of limestone hills; ca. 100 m elev.

#### Etymology.

The specific epithet reflects the morphology of the inner petals.

#### Local name.

Karawek Phruksa Sawan.

#### Notes.

The fruit and seeds are only known from fresh material and have not been preserved due to fungal infection.

The distinction between *A. spathulatus* and *A. tanaosriensis* is corroborated by unpublished phylogenetic analysis of combined chloroplast (*matK*, *ndhF*, *psbA-trnH*, *trnL-F*) and nuclear (*AP3*, *carboxymethylenebutenolidase*, *GI*, *HMGS*, *LFY*, *mag1*, *ncpGS*, *NIA*, *PhyA*, *RPB2*) DNA sequence data, which retrieved *A. spathulatus* as sister to *A. uniflorus*, with *A. tanaosriensis* sister to *A. suaveolens* (J. Chen et al. unpublished data).

##### New records for Thailand

### Artabotrys byrsophyllus

Taxon classificationPlantaeMagnolialesAnnonaceae

I.M.Turner & Utteridge

Fig. 3

Artabotrys byrsophyllus I.M.Turner & Utteridge, Nordic J. Bot. 33: 562 (2015). – TYPE: Malaysia, Peninsular Malaysia, Kelantan, Ulu Sungei Aring near Kuala Tapah, 21 Sep 1967, *P.F. Cockburn FRI 7151* (holotype: K! [2 sheets, barcodes K 000607815, K 000607816], isotype: KEP, n.v.).

#### Distribution and habitat.

Northern Peninsular Malaysia (Kedah, Kelantan, and Trengganu) and southern Thailand (Narathiwat and Songkhla), in lowland and hill rain forests (Fig. 5).

**Figure 3.**
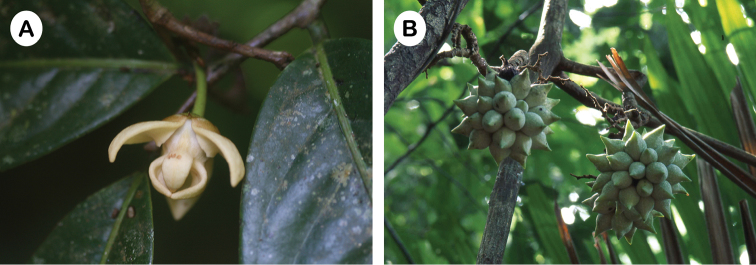
*Artabotrys byrsophyllus*. **A** Flower **B** Fruit (Photos: P. Chalermglin).

#### Local name.

Karawek Bai Yai.

#### Specimens examined.

Thailand: Narathiwat Province, Kaluwotai, Khao Chana, 21 Sep 1985, *C. Niyomdham et al.* 1065 (AAU, BKF); Waeng district, *T. Insura 77* (KUFF); idem, *T. Insura 78* (KUFF); idem, *T. Insura 90* (KUFF); idem, *A. F. G. Kerr 14474* (BM); idem, *S. Phusomsaeng 389* (BKF).

#### Notes.

The name *A. grandifolius* is misapplied by Chalermglin (2001), Thongpairoj (2008, as “*A. grandiflorus*”) and Insura (2009), for which the name *A. byrsophyllus* should be applied; *A. grandifolius* sensu King is restricted to Peninsular Malaysia. *Artabotrys byrsophyllus* can be distinguished from *A. grandifolius* by reference to its leathery leaves, reticulate tertiary leaf venation that is indistinct on both surfaces, shorter petals (outer petals 13–16 mm vs. ca. 21 mm; blade of inner petals ca. 8 mm vs. 16–17 mm) and subsessile monocarps with marked apiculum.

### Artabotrys punctulatus

Taxon classificationPlantaeMagnolialesAnnonaceae

C.Y.Wu ex S.H.Yuan

Fig. 4

Artabotrys punctulatus C.Y.Wu ex S.H.Yuan, Acta Bot. Yunnan. 4: 260 (1982). – TYPE: China, Yunnan, Jinghong, 1015 m, 9 Apr 1957, *Exped. Biol. Sino-Ross. ad prov. Yunnan* 7654 (holotype: KUN! [barcode KUN 0045889]; isotypes: IBSC, n.v., PE! [barcode PE 00934500]).

#### Distribution and habitat.

Southern China (Yunnan) and Thailand (Khao Yai National Park and Phu Kradueng National Park), in lower montane forests, 1000–1500 m elev. (Fig. 5).

**Figure 4.**
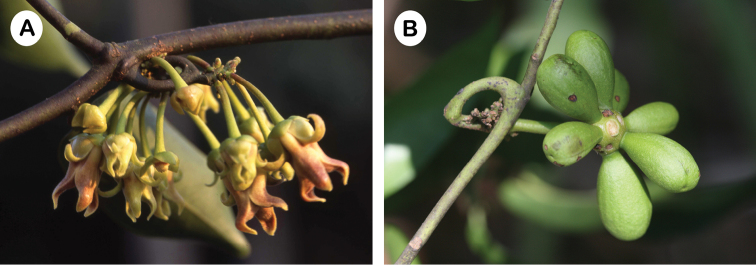
*Artabotrys punctulatus*. **A** Inflorescence **B** Fruit (Photos: P. Chalermglin).

**Figure 5.**
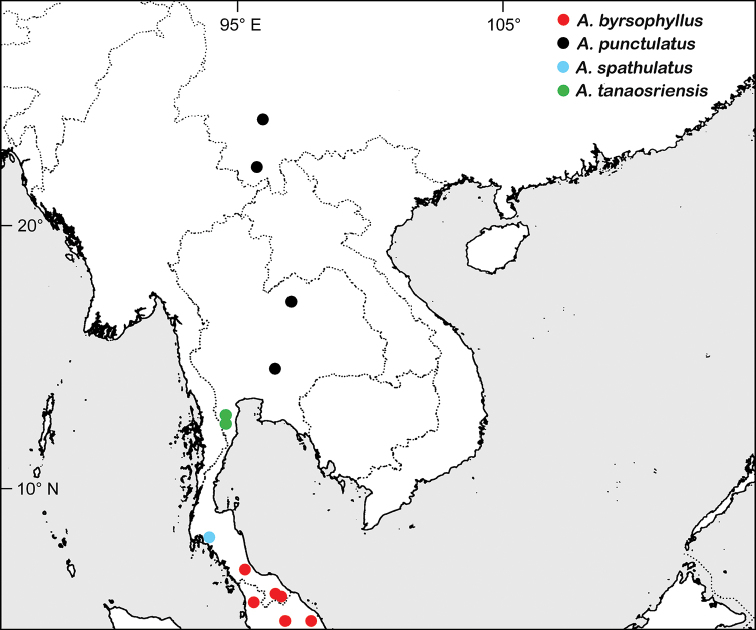
Distributions of *A. byrsophyllus*, *A. punctulatus*, *A. spathulatus* and *A. tanaosriensis* in Thailand and surrounding areas. Localities of *A. byrsophyllus* in Peninsular Malaysia were retrieved from Turner and Utteridge (2015). Localities of *A. punctulatus* in Yunnan were retrieved from georeferenced herbarium records in GBIF (https://www.gbif.org/).

#### Local name.

Karawek Kra.

#### Specimens examined.

Thailand: Loei Province, Phu Kradueng National Park, 24 Dec 1971, 1300 m, *van Beusekom et al. 4555* (L); Nakhon Ratchasima Province, Pak Chong District, Khao Yai National Park, 1200 m, *T. Insura 43*; idem, *T. Insura 101*; idem, *T. Insura 102*; idem, *T. Insura 103*; idem, *T. Insura 104* (KUFF); idem, *U. Thongpairoj 235* (CMU).

#### Notes.

Specimens of this species were mistakenly recognised as a new species (“*A.* sp. 5 (Kao Yai)”) by Thongpairoj (2008) and misidentified as “*A. aereus*” by Insura (2009). *Artabotrys punctulatus* can be distinguished from *A. aeneus* (which is only known from Vietnam) by its chartaceous leaves, sparsely pubescent (vs. densely villose) flowering pedicels, sepals and petals, smaller sepals and its elongated, raised rim between the claw and blade on the adaxial surface of the inner petals.

##### Key to the native *Artabotrys* species in Thailand

**Table:** 

1	Young branches velutinous; abaxial surface of leaves densely hairy	**2**
–	Young branches glabrous to sparsely hairy; abaxial surface of leaves glabrous to sparsely hairy	**3**
2	Leaf apex acuminate; leaf laminas broadly elliptic and coriaceous; petal blades 10–15 mm wide	***A. siamensis***
–	Leaf apex caudate; leaf laminas oblong and chartaceous; petal blades 2–5 mm wide	***A. uniflorus***
3	Pedicels less than a quarter of the length of the flower	**4**
–	Pedicels slightly shorter, as long as, or longer than flower	**6**
4	Petal blades linear, 1–2 mm wide; sepals ca. 3 mm long, ca. 2.5 mm wide; leaf laminas 10.5–19 cm long, 4–9 cm wide	***A. tipuliferus***
–	Petal blades oblong-elliptic, 7–12 mm wide; sepals 4–10 mm long, 4–8 mm wide; leaf laminas 7–14 cm long, 2.5–4.2 cm wide	**5**
5	Inflorescences with up to 5 flowers; petal blades chartaceous, 2.8–3.3 cm long, 0.7–1 cm wide; monocarps distinctly stipitate (stipes 3–5 mm long) and slightly beaked (beak 1–2 mm long)	***A. brevipes***
–	Inflorescences with a solitary flower; petal blades coriaceous, 3.5–4 cm long, ca. 1.2 cm wide; monocarps sessile and sharply beaked (beak up to 1 cm long)	***A. oxycarpus***
6	Stamen connectives apiculate	**7**
–	Stamen connectives truncate	**8**
7	Young branches often with spines; leaf apex retuse, truncate or mucronate; leaf laminas 6–8 cm long, 2.5–4.5 cm wide; monocarps ellipsoid, 3.5–4.5 cm long, 1–2 cm wide	***A. spinosus***
–	Young branches without spines, leaf apex acuminate; leaf laminas 9–15 cm long, 3–7 cm wide; monocarps obovoid, 3–3.5 cm long, 2–2.5 cm wide	***A. harmandii***
8	Petal blades terete; monocarps 1–2(–4) per fruit	***A. suaveolens***
–	Petal blades not terete; monocarps 4–30 per fruit	**9**
9	Inflorescences with 10–20 flowers; monocarps not beaked	**10**
–	Inflorescences with up to 4 flowers; monocarps beaked	**11**
10	Petals chartaceous, yellow (in fresh material); adaxial surface of inner petals with a short, raised rim between claw and blade (ca. 1 mm long); leaf laminas coriaceous	***A. multiflorus***
–	Petals coriaceous, green to beige with maroon patches (in fresh material); adaxial surface of inner petals with an elongated, raised rim between claw and blade (ca. 5 mm long); leaf laminas chartaceous	***A. punctulatus***
11	Outer petals with externally distinct blades and claws; inner petal blades rhomboid or spathulate	**12**
–	Outer petals without externally distinct blades and claws; inner petal blades ovate or triangular	**13**
12	Outer petal blades undulate; outer petal claws broadly ovate; inner petal blades rhomboid and undulate; monocarps slightly beaked (beak less than 1 mm long)	***A. tanaosriensis***
–	Outer petal blades flat; outer petal claws broadly rhomboid; inner petal blades broadly spathulate and strongly concave; monocarps strongly beaked (beak ca. 2 mm long)	***A. spathulatus***
13	Leaf laminas chartaceous, 3–4 cm wide; leaf base asymmetrical; flowering pedicels 4–6 mm long; monocarps 2–2.5 cm long, 0.8–1.2 cm wide	***A. oblanceolatus***
–	Leaf laminas coriaceous, 4–13.5 cm wide; leaf base symmetrical; flowering pedicels 10–25 mm long; monocarps 3–4.5 cm long, 2–3.5 cm wide	**14**
14	Leaf laminas 9–15.5 cm long, 4–7 cm wide; outer petals 1.5–2 cm long; monocarps ca. 6 per fruit, 4–4.5 cm long, 2–3.5 cm wide	***A. venustus***
–	Leaf laminas 21–32 cm long, 8.5–13.5 cm wide; outer petals 1.3–1.6 cm long; monocarps ca. 20 per fruit, 3–3.5 cm long, ca. 2 cm wide	***A. byrsophyllus***

## Supplementary Material

XML Treatment for Artabotrys tanaosriensis

XML Treatment for Artabotrys spathulatus

XML Treatment for Artabotrys byrsophyllus

XML Treatment for Artabotrys punctulatus
